# IncF plasmid diversity in multi-drug resistant *Escherichia coli* strains from animals in China

**DOI:** 10.3389/fmicb.2015.00964

**Published:** 2015-09-22

**Authors:** Qiu-E. Yang, Jian Sun, Liang Li, Hui Deng, Bao-Tao Liu, Liang-Xing Fang, Xiao-Ping Liao, Ya-Hong Liu

**Affiliations:** ^1^Laboratory of Veterinary Pharmacology, College of Veterinary Medicine, South China Agricultural UniversityGuangzhou, China; ^2^Jiangsu Co-Innovation Centre for Prevention and Control of Important Animal Infectious Diseases and ZoonosesYangzhou, China

**Keywords:** *Escherichia coli*, IncF plasmid, multi-resistance, addiction systems, RFLP

## Abstract

The purpose of this study was to characterize a collection of 103 multidrug resistance IncF plasmids recovered from *Escherichia coli* of food producing and companion animals between 2003 and 2012. A total of 103 incF plasmids were characterized using an established PCR-based IncF replicon sequence typing (RST) system to identify FII, FIA, and FIB (FAB) groups. Plasmids were also analyzed using-restriction fragment length polymorphism (RFLP). Antibiotic Resistance determinants *bla*_CTX-*M*_, plasmid-mediated quinolone resistance (PMQR) genes and *rmtB* and plasmid addiction systems (PAS) were identified by PCR screening. A total of 20 different RSTs from 103 IncF plasmids were identified. The groups F2 and F33 with the RST formulae A-: B- were the most frequently encountered types (63.1%). The antibiotic resistance genes (ARGs) *bla*_CTX-*M*_, *rmtB*, and *oqxB* were carried by 82, 37, and 34 IncF plasmids, respectively. Most of these plasmids carried more than one resistance gene (59.2%, 61/103). The IncF plasmids also had a high frequency of addiction systems (mean 2.54) and two antisense RNA-regulated systems (*hok–sok* and *srnBC*) and a protein antitoxin-regulated system (*pemKI*) were the most prevalent. Not surprisingly, RFLP profiles among the IncF plasmids were diverse even though some shared identical IncF-RSTs. This is the first extensive study of IncF plasmid-positive *E. coli* isolates from animals in China. Our results demonstrate that IncF is the most prevalent plasmid family in *E. coli* plasmids and they commonly carry multiple resistance determinants that render them resistant to different antibiotic classes simultaneously. IncF plasmids also harbor addiction systems, promoting their stability and maintenance in the bacterial host, under changing environmental conditions.

## Introduction

Increasing antibiotic resistance in bacteria has potentially disastrous consequences for human and animal health around the world. Recent studies have demonstrated that plasmids act as efficient vehicles for the spread of antibiotic resistance genes (ARGs) more frequently than previously believed (Taylor et al., 2004; García-Fernández et al., 2009; Dolejska et al., 2011; Accogli et al., 2013; Carattoli, 2013; Dahmen et al., 2013b). Thus far, 27 different plasmid incompatibility (Inc.) groups are recognized in the Enterobacteriaceae (Couturier et al., 1988; Carattoli et al., 2005; Villa et al., 2010). A smaller number of particular plasmid families play a major role in the diffusion of specific resistance genes among Enterobacteriaceae and are the most frequently encountered. The IncF plasmids represent one of the most prevalent incompatibility types, and have been identified worldwide in Enterobacteriaceae from different origins and sources (Carattoli, 2009; Mathers et al., 2015). Currently, a total of 924 plasmids have been deposited at the plasmid multilocus sequence typing (pMLST) database (http://pubmlst.org/plasmid/, 30 January, 2015). Of these, 214 plasmids belong to IncF group and 158 of them (73.8%) recovered from *Escherichiacoli* could be classified in 90 different subgroups on the basis of their FII, FIA and FIB (FAB) alleles.

IncF plasmids have systems which guarantee their autonomous replication but also encode addiction systems frequently based on toxin-antitoxin factors. These systems contribute to control their copy number and ensurethe instable inheritance during cell division (Cohen, 1976; Kado, 1998; Hayes, 2003). In our previous studies, we found that IncF plasmids could integrate a wide range of genes conferring resistance to all major classes of antimicrobials including β-lactams, aminoglycosides, tetracyclines, chloramphenicol, and quinolones (Liao et al., 2013; Liu et al., 2013). The extended-spectrum β-lactamases (ESBLs), especially those of the CTX-M type, are often associated with plasmid-mediated quinolone resistance (PMQR) and aminoglycoside resistance genes. Plasmids belonging to the IncF group have been the primary hosts for these traits (Boyd et al., 2004; Yao et al., 2011; Matsumura et al., 2013; Ogbolu et al., 2013).

The spread of such multidrug resistance plasmids among Enterobacteriaceae strains could also affect clinical treatments. For instance, IncF-*bla*_CTX-*M*_ association was observed from both human and animal *E. coli* isolates such as the IncF-like plasmid R100 involved in the dissemination of *bla*_CTX-*M*-14_ in Hong Kong, United Kingdom and France (Woodford et al., 2009; Ho et al., 2012; Dahmen et al., 2013a). The genes *rmtB, qepA, qnr, fosA3*, and *oqxAB* have been recently identified on IncF plasmids in *E. coli* from China, Korea and Spain (Tamang et al., 2008; Li et al., 2012; Ruiz et al., 2012; Ho et al., 2013). All together, the IncF-related family has the potential to be a major contributor worldwide to the diffusion of different resistance genes.

In general, the IncF plasmids are not a homogeneous group and vary in size (50–200 kb) and replicon type (Garcillán-Barcia et al., 2009; de Been et al., 2014; Lanza et al., 2014). Most importantly, plasmids encoding virulence-associated traits fall almost exclusively within IncF group (Johnson and Nolan, 2009). Therefore, knowledge of the prevalence and IncF plasmid subtypes in resistant populations could be a useful aid in exploring novel plasmid-targeting strategiesto treat resistant bacteria (Osborn et al., 2000; DeNap and Hergenrother, 2005; Baquero et al., 2011). We investigated variations in IncF plasmids and the phylogenetic relationship among IncF plasmids from different sources using RST and RFLP analyses. This study characterized IncF plasmids according to resistance genes, genetic relatedness and addiction systems in 103 *E. coli* isolates from pigs, poultry and companion animals.

## Materials and methods

### Transconjugants and antibiotic susceptibility testing

A total of non-duplicate 795 *E. coli* were isolated from animals between 2002 and 2012, according to the data published in our previous study (Liao et al., 2013; Liu et al., 2013, 2014). Among them, 696 *E. coli* were isolated from liver, heart or faces samples of diseased food-producing animals including 318 from avian (177 from ducks, 110 from chicken and 31 from geese) and 378 from pigs. The food-producing animals originated from more than 80 farms all over Guangdong province. The remaining 99 isolates were from rectal swab samples of pets including 71 dogs and 28 cats in five animal hospitals in Guangdong province. Further information about these animals, the underlying disease and possible antimicrobial pretreatment were unfortunately not available. One suspected colony with typical *E. coli* morphology and size was selected from all agar plates of each sample and was identified by Matrix Assisted Laser Desorption/lionization Time-of-Flight mass spectrometry (MALDI-TOF VITEK MS RUO System) using the Saramis™ database (bioMérieux, France). The genetic relatedness among partial *E. coli* isolates randomly selected were determined by pulsed-field gel electrophoresis (PFGE) with *XbaI* according to a protocol described previously (Gautom, 1997). Isolates encoding *bla*_CTX−M_, *rmtB* and/or PMQR gene (535/795), were selected for conjugation experiments by the broth-mating method using streptomycin-resistance *E. coli* C600 as the recipient. Transconjugants were selected on MacConkey agar plates containing streptomycin (1000 mg/L) and cefotaxime (2 mg/L)/amikacin (32 mg/L)/olaquindox (64 mg/L), respectively. The transconjugants harboring *bla*_CTX-*M*_, *rmtB*, and/or PMQR gene mentioned above were confirmed by PCR as previously.

One hundred and three transconjugants, each carrying one single IncF plasmid, were selected from a collection of clonally unrelated *E. coli* strains (15 pets, 43 avian, and 45 pigs) in our study. Table S1 contains detailed information for this population. Within this group 28 IncF plasmids had been previously characterized (Liao et al., 2013; Liu et al., 2013, 2014). Antimicrobial susceptibility tests were determined by agar dilution method on Mueller-Hinton agar plates. The antimicrobial drugs studied included quinolones (nalidixicacid), fluoroquinolones (ciprofloxacin, enrofloxacin, and levofloxacin), ampicillin, third-generation cephalosporins (ceftiofur, cefotaxime and ceftazidime), aminoglycoside (streptomycin, amikacin, kanamycin, and gentamycin) and other antimicrobials (olaquindox, chloramphenicol, florfenicol, and tetracyclines). The susceptibility tests were carried out and evaluated according to the guidelines of the Clinical and Laboratory Standards Institute (CLSI). The breakpoints for each antimicrobial were those recommended by the CLSI (M100-S19), CLSI (Vet01-A4/Vet01-S2), and DANMAP 98 (olaquindox) (Clinical and Laboratory Standards Institute, 2008, 2013). *E. coli* ATCC 25922 was used for quality control purposes.

### Detection of antimicrobial resistance genes

The total DNA of transconjugants harvested from LB broth were extracted by TIANPrep Plasmid Mini Kit (TIANGEN Biotech, Beijing, China), according to the manufacturer's instructions. The *bla*_CTX-*M*_, *rmtB*, and PMQR genes were screened by PCR using primers as described previously (Cano et al., 2009; Wang et al., 2012). The *bla*_CTX-*M*_, *rmtB*, and *oqxB*-positive transconjugants were also evaluated for the presence of other PMQR genes (*qnrA, qnrB, qnrC, qnrD, qnrS*, and *qepA*). PCR products were sequenced and compared with the reported sequences from GenBank (www.ncbi.nlm.nih.gov/genbank/).

### IncF plasmid analysis

Plasmid DNA was extracted by QIAGENPrep Plasmid Midi Kit (QIAGEN, Hilden, Germany), according to the manufacturer's instructions. PCR-base replicon typing (PBRT) was performed on all transconjugants carrying a single plasmid, as previously described (Carattoli et al., 2005). To better characterize IncF, replicon sequencing typing (RST) was performed according to protocols described previously (Villa et al., 2010), and alleles were assigned by comparing the amplicon sequence to the plasmid MLST database (http://pubmlst.org/plasmid/).

Plasmid sizes in the transconjugants were determined by pulsed-field gel electrophoresis using nuclease S1 (TaKaRa Biotechnology, Dalian, China) digestion of plasmid DNA prepared in agarose blocks (S1-PFGE). DNA fragments were separated by PFGE on a CHEF-DR III apparatus (Bio-Rad, Hercules, CA, USA) for 15 h at 6 V/cm at 14°C with an initial pulse time of 1 s and a final pulse time of 12 s. Southern hybridization was performed following S1-PFGE using *bla*_CTX-*M*_, *rmtB*, and *oqxB*-specific digoxigen in (DIG)-labeled probes. Hybridization was detected using the DIG Nucleic Acid Detection Kit (Roche Diagnostics). All plasmids were also analyzed by restriction fragment length polymorphism (RFLP) as following: Plasmid DNA was digested with *EcoRI* and electrophoresed in 1.0% agarose at 5 V/cm for 2 h. DNA was visualized by EtBr staining. Cluster analysis of digestion patterns generated dendrograms and plasmid similarity was measured using BioNumerics software (Applied Maths, Sint-Martens-Latem, Belgium) using the Dice Similarity Index (DSI) (tolerance 1.5% and optimization 1.5%. Plasmids with a DSI ≥80% were assigned to the same cluster (designated with numbers 1, 2, 3, and 4). Letters were used to discriminate RFLP patterns assigned to the same clusterthat differed by one or two restriction bands (i.e., 1a, 1b, 1c etc.).

### Identification of plasmid addiction systems

Plasmid-mediated addiction systems were identified using primers and amplification conditions were previously described (Mnif et al., 2010). We screened for 8 major addiction systems: *ccdA*-*ccdB* (involved in cell division), *pemK*-*pemI* (for plasmid emergency maintenance), *relB*-*relE* (relaxed control of stable RNA), *parD*-*parE* (DNA replication), *vagC*-*vagD* (virulence-associated protein), *hok-hok* (host-killing), *snrB*-*snrC* (RNA stability), and *pndA*-*pndC* (promotion of nucleic acid).

### Statistical analysis

Statistical significance for the comparison of prevalence data and proportions was determined by the *x*^2^-test. *p*-values less than 0.05 were deemed to be statistically significant.

## Results

### Antibiotic resistance phenotypes

A total of 103 transconjugants were examined in this study and 96% exhibited multi-drug resistance phenotypes with to ampicillin and streptomycin. More than half of the isolates also showed resistance tocefotaxime, ceftriaxone, gentamicin, kanamycin and ceftiofur (Table S1). The antimicrobial resistance rates to other antibiotics tested as follows: amikacin (43.7%), olaquindox (42.7%), tetracycline (40.8%), chloramphenicol (34.0%), and florfenicol (26.2%). Most of these isolates were still susceptible to levofloxacin, enrofloxacin and ciprofloxacin.

### Detection of resistance genes

Results of screening for resistance genes among 103 transconjugants are shown in Table S1. The predominant resistance genes identified were *bla*_CTX-*M*_, *rmtB*, and *oqxB* found in 82, 37, and 34 IncF-type plasmids, respectively. Interestingly, there was a range of *bla*_CTX-*M*_ genotypes including M55 (*n* = 30), M14 (*n* = 20), M27 (*n* = 14), M65 (*n* = 13), M15 (*n* = 3) and M24 and M3 (1 each). A smaller subset also harbored *qnrS, qepA*, and *qnrB* genes (11, 6, and 3, respectively). Sixty-one plasmids carried more than one resistance gene, and the most prevalent combinations were *bla*_CTX-*M*_-*rmtB* (*n* = 28) and *bla*_CTX-*M*_-*oqxAB* (*n* = 15). The *qnrA, qnrC*, and *qnrD* loci were not found in any of the plasmids tested.

### Characterization of IncF plasmids

S1-PFGE and Southern blot hybridization analysis showed that all the 103 multi-drug resistant strains contained plasmids ranging in size from 60 to 194 kb (Table 1). This group contained very diverse IncF-RST patterns. A total of 20 IncF-RSTs were found that had replicon FII alone or in combination with FIA or FIB. Various alleles were observed for each of FII (F2, F14, F16, F18, F31, F33, F35, F36, F43, F46, and F58), FIA (A-, A1, A3, and A4) and FIB (B-, B1, B8, B10, and B24) replicons. Meanwhile, we found identical combinations were detected in a number of different plasmids; F2: A-: B- (*n* = 35) and F33: A-: B- (*n* = 30) were the most common. Curiously, more than half of F2: A-: B- positive *E. coli* strains (54.8%, 19/35) were recovered from pigs, while F33: A-: B- (46.7%, 14/30) and F18: A-: B- (50.0%, 5/10) were mainly found in *E. coli* strains recovered from avians.

**Table 1:** **The distribution of addiction systems and resistance genes according to FAB type identified in the 103 multi-resistance plasmids**.

**FAB**	**No. (size: kb)**	**Addiction systems (No.)**							**Mean**	^*^Resistance genes (No.)											
		***hok-sok***	***pemKI***	***snrBC***	***vagCD***	***ccdAB***	***pndAC***	**Total**		**M55**	**M14**	**M27**	**M65**	**M15**	**M3**	**M24**	***rmtB***	***oqxB***	***qnrB***	***qnrS***	***qepA***
F2:A-:B-	35(70~194)	28	18	0	3	14	3	66	1.9	5	8	14	2	0	1	1	16	11	3	5	6
F2:A1:B-	3(120~145)	3	0	3	3	0	0	9	3	1	2	0	0	0	0	0	0	0	0	1	0
F2:A1:B1	2(145~194)	2	1	2	2	2	0	9	4.5	0	0	0	0	0	0	0	2	1	0	1	0
F2:A-:B10	1(~120)	1	0	1	1	0	0	3	3	0	1	0	0	0	0	0	0	0	0	0	0
F14:A-:B-	1(ND)	0	1	0	1	0	0	2	2	0	1	0	0	0	0	0	0	1	0	0	0
F16:A-:B-	3(~97)	3	1	1	0	0	0	5	1.7	0	0	0	0	0	0	0	0	3	0	2	0
F16:A-:B1	1(~60)	1	1	0	0	1	0	3	3	1	0	0	0	0	0	0	0	0	0	0	0
F18:A-:B-	3(135~145)	3	3	3	0	0	0	9	3	1	1	0	1	0	0	0	0	1	0	0	0
F18:A-:B1	1(145~194)	8	8	9	1	2	1	29	2.9	5	0	0	0	0	0	0	1	6	0	0	0
F18:A-:B8	1(~194)	1	0	1	0	1	0	3	3	0	0	0	0	0	0	0	0	1	0	0	0
F31:A4:B1	3(~194)	1	3	3	2	3	0	12	4	0	0	0	0	3	0	0	0	0	0	0	0
F33:A-:B-	30(60~194)	26	25	29	1	1	2	84	2.8	16	0	0	9	0	0	0	17	8	0	0	0
F33:A1:B-	1(ND)	1	0	0	1	1	0	3	3	0	1	0	0	0	0	0	0	0	0	0	0
F33:A-:B1	1(ND)	1	1	1	0	0	0	3	3	0	0	0	0	0	0	0	1	1	0	0	0
F35:A-:B-	3(~97)	3	0	3	2	1	0	9	3	0	3	0	0	0	0	0	0	0	0	0	0
F36:A1:B1	1(~160)	1	1	1	0	1	0	4	4	0	1	0	0	0	0	0	0	0	0	0	0
F43:A-:B-	1(~110)	1	0	0	0	1	0	2	2	0	1	0	0	0	0	0	0	0	0	0	0
F43:A3:B-	1(~145)	1	1	1	1	1	0	5	5	1	0	0	0	0	0	0	0	0	0	1	0
F46:A-:B24	1(~130)	1	0	1	0	0	0	2	2	0	0	0	0	0	0	0	0	1	0	1	0
F58:A-:B-	1(~145)	0	0	0	0	0	0	0	0	0	1	0	1	0	0	0	0	0	0	0	0
Total No.	103	86	64	59	18	29	6	262	2.54	30	20	14	13	3	1	1	37	34	3	11	6

* M55/14/27/65/15/3/24 is resistance genes abbreviations for bla_CTX−M−55∕14∕27∕65∕15∕3∕24_, respectively.

ND, not determined.

Further analysis of IncF plasmids bearing multi-resistance genes was performed to see whether a particular resistance gene was associated with a specific plasmid backbone defined by replicon type and addiction system content. Notably, there was higher prevalence of resistance genes located on F2: A- B- and F33: A-: B- plasmids than on the remaining replicon types (Table 1). For instance, *bla*_CTX-*M*-27_, *qepA*, and *qnrS* were preferentially associated with the F2: A-: B-. The *bla*_CTX-*M*-55_, which was the most common identified β-lactamase in our study, combined with F33: A-: B-; while *rmtB* and *oqxB* were mainly found in F2: A-: B- and F33: A-: B-.

**Figure 1:**
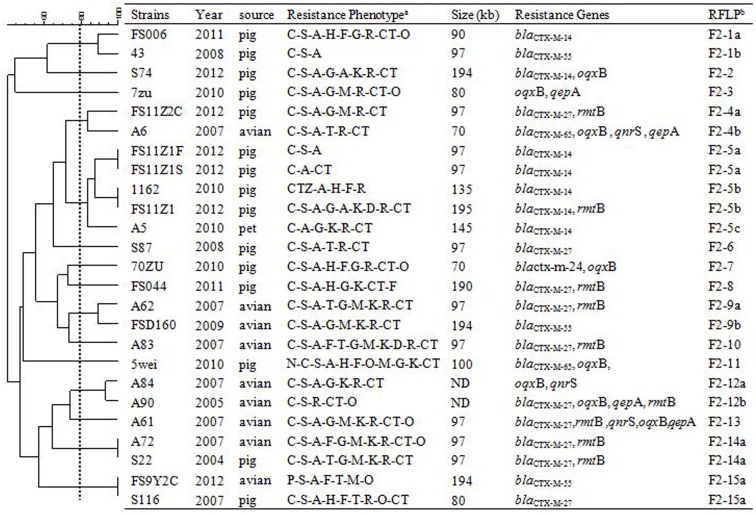
**RFLP patterns and characterization of multi-resistance 25 F2: A-: B- plasmids**.

**Figure 2:**
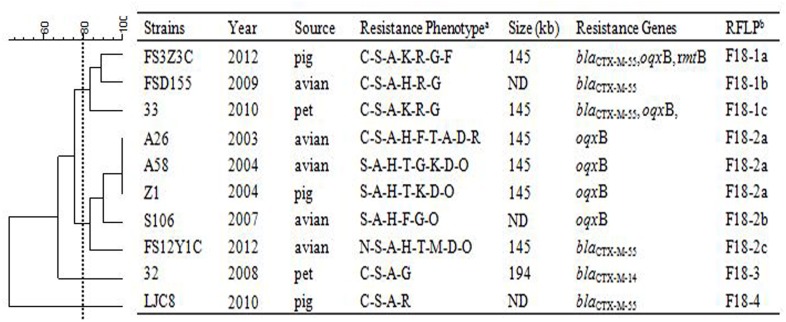
**RFLP patterns and characterization of multi-resistance 10 F18: A-: B1 plasmids**.

**Figure 3:**
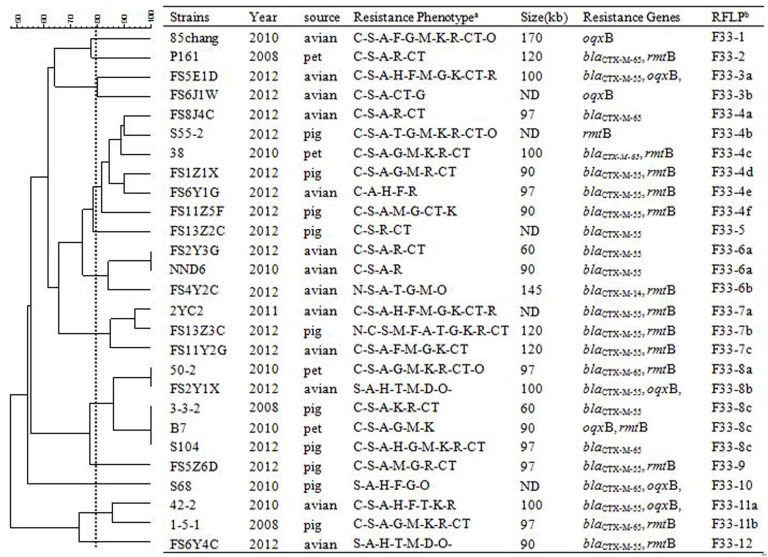
**RFLP patterns and characterization of multi-resistance 27 F33: A-: B- plasmids**. The scale on the top left of the figure indicates the percentage of similarity for the *EcoR*I restriction profiles of the IncF plasmids. ^a^Antimicrobial for which the plasmids MICs fell within the resistant range; Antimicrobial abbreviations: A, ampicillin; S, streptomycin; M, amikacin; K, kanamycin; G, gentamycin; R, ceftriaxone; C, cefotaxime; CT, ceftiofur; N, nalidixic acid; P, ciprofloxacin; E, enrofloxacin; L, levoflox-acin; O, olaquindox; T, tetracycline; H, chloramphenicol and F, florfenicol. ^b^Plasmids with a DSI ≥80% were assigned to the same cluster (designated with numbers 1, 2, 3, 4 etc.). Letters were used to discriminate RFLP patterns assigned to the same cluster, but differing by one or two restriction bands (i.e., 1a, 1b, 1c etc.). ND, not determined.

In order to assess relationships among the different replicon types of 103 IncF plasmids encoding variable resistance determinants, RFLP analysis was conducted which allowed visualization of variable clustering. However, our RFLP results showed diverse plasmid profiles even among identical IncF-RST groups. For instance, among 27/30 F33: A-: B- and 25/35 F2: A-: B- plasmids, 12 and 15 RFLP patterns were identified, respectively. In addition, antimicrobial susceptibility, plasmid size and antimicrobial resistance genes varied (Figures 1–3).

### Addiction systems

Of the eight addiction systems, the most frequently represented systems were *hok-sok* (*n* = 86), followed by *pemKI* (*n* = 64), *snrBC* (*n* = 59), *ccdAB* (*n* = 29), *vagCD* (*n* = 18), and *pndAC* (*n* = 6). None of the IncF-type plasmids harbored *parDE* and *relBE*. The average mean of addiction system was 2.54 with a range of 0–5 per plasmid. Interestingly, when the replicon FII was associated with FIA and FIB, the isolate also carried more addiction systems than in the absence of this association (Table 1). For instance, between the groups F2: A1: B1 and F2: A-: B-, there was a mean of 4.5 vs. 1.9, and the combination of F16: A-: B1 and F16: A-: B- with the mean of 3.0 vs. 1.7.

The number of different types of addiction systems also varied between the most prevalence replicon types in this study, F2: A-: B- and F33: A-: B-. The number of *snrBC* loci on F33: A-: B- was significantly higher than on F2: A-: B- (*P* < 0.01). In contrast, this number was reversed in the case of the *ccdAB* system (Table 1).

## Discussion

In the present study, the prevalence of the resistance determinants *bla*_CTX-*M*_, PMQR genes and *rmtB* in IncF plasmids from farm animals and pets in China was investigated and the characteristics of IncF plasmids were elucidated. One-hundred and three IncF plasmids were recovered, the majority from pigs and avians (85.4%, 88/103), and the remaining obtained from pets (14.6%, 15/103), demonstrating that IncF plasmids are prevalent among multi-drug resistant *E. coli* strains from animals in China. The IncF plasmids identified in our study were associated with multidrug resistance profiles that conferred resistance to different classes of antimicrobials simultaneously. Most of IncF plasmids harbored *bla*_CTX-*M*_ and, frequently carried other resistance genes including *qnr, qepA, oqxB*, and *rmtB*. These results are in agreement with previous findings that the *rmtB* and PMQR genes are often linked with *bla*_CTX-*M*_-located on the same plasmid (Dhanji et al., 2011; Li et al., 2012; Matsumura et al., 2013; Chen et al., 2014). Together, these data suggest that the spread of multiple ARGs on the same plasmid has been an important dissemination mechanism of multidrug resistance (Liebert et al., 1999).

The most impressive finding in this study was the diversity of IncF plasmids. The result is similar to previous reports that showed IncF plasmids are not a homogeneous group (Osborn et al., 2000; Coque et al., 2008; Villa et al., 2010; Shin et al., 2012). Moreover, heterogeneous RFLP patterns among identical IncF-RST groups indicate that the cargos such as resistance genes are also likely associated with the diversity of IncF plasmids. Interestingly, we identified some particular resistance genes were associated with the specific plasmid backbone defined by replicon type. For instance, *bla*_CTX-*M*-3_ was located on F2: A-: B-, whereas it has been previously on detected IncL/M plasmids (Golebiewski et al., 2007). As for *bla*_CTX-*M*-14_, which was primarily found on F2: A-: B- in our study, but in Spain and France, this gene was presented on the IncK plasmids (Diestra et al., 2009; Valverde et al., 2009). Although the movements of these resistance genes were attributed to transposable elements, it is certain that the epidemic plasmids may accelerate their widespread dissemination.

Interestingly, although RFLP patterns were diverse among the plasmids, some with the same IncF-RST grouping did show similar RFLP patterns with minor variations. These data agree with previous studies reporting that IncF plasmids form a very heterogeneous group (Dahmen et al., 2013b). Thus, the extensive diversity of RFLP patterns among IncF plasmids from the collection of *E. coli* used in this study may be due to frequent recombination and acquisition resistance genes between the plasmids (Partridge et al., 2011; Wiedenbeck and Cohan, 2011; Cain and Hall, 2012).

In contrast to RFLP patterns, the content of addiction systems seemed to differ by replicon type. For instance, the *snrBC* system was widely represented in F33: A-: B- but absent in F2: A-: B-, while *ccdAB* was associated with F2: A-: B-. This finding indicates a linkage between addiction systems and specific plasmid backbones, but this must be investigated further. As previously reported, plasmid addiction systems (PAS) contribute to the stability and maintenance of plasmids and they have been shown to facilitate plasmid dissemination even in the absence of antibiotic selection (Hayes, 2003; Moritz and Hergenrother, 2007). Together, these data suggest a hypothesis that the diversity of IncF plasmids can be driven by a combination of acquired multiple resistance genes and addiction modules. In this way, host maintenance would be facilitated by their spread in the *E. coli* population.

In conclusion, we identified extensive diversity of RSTs, plasmid sizes, addiction systems and RFLP patterns among IncF plasmids, carrying *bla*_CTX-*M*_ or PMQR or aminoglycoside resistance genes. Considering the fact that the IncF plasmids have been common reservoirs for diffusion of resistance genes, its wide dissemination among Enterobacteriaceae especially among *E. coli* could pose a serious threat to public health.

### Conflict of interest statement

The authors declare that the research was conducted in the absence of any commercial or financial relationships that could be construed as a potential conflict of interest.
