# Supplementary material for: IncF plasmid diversity in multi-drug resistant Escherichia coli strains from animals in China
Source: Front Microbiol. 2015 Sep 22;6:964. doi: 10.3389/fmicb.2015.00964 (PMC4585273; doi:10.3389/fmicb.2015.00964)
Supplement: Supplementary file 1 [file Table1.DOCX]

**Supplementary data**

**IncF plasmid diversity in multi-drug resistant *Escherichia coli* strains from animals in China**

Table S1. Characteristics of 103 Multi-resistance IncF Plasmids

| FAB^a^ | Isolate | Year | Source | Co-resistance^b^ | Size^c^ | Genes^d^ | Reference |
| --- | --- | --- | --- | --- | --- | --- | --- |
| F2:A-:B- | FS006 | 2011 | pig | C-S-A-L-F-G-R-CT-O | 90 | M14 | this study |
| F2:A-:B- | S74 | 2012 | pig | C-S-A-G-K-N-R-CT | 194 | M14*, oqxB* | this study |
| F2:A-:B- | A5 | 2010 | pet | C-A-G-N-R-CT | 145 | M14 | Liu,B.T. et al. |
| F2:A-:B- | FS11Z1 | 2012 | pig | C-S-A-G-K-N-D-R-CT | 195 | M14*, rmtB* | this study |
| F2:A-:B- | FS11Z1S | 2012 | pig | C-A-CT | 97 | M14 | this study |
| F2:A-:B- | FS11Z1F | 2012 | pig | C-S-A | 97 | M14 | this study |
| F2:A-:B- | FS4Z6S | 2012 | pig | C-S-A-N-CT | 145 | M14 | this study |
| F2:A-:B- | 1162 | 2010 | pig | C-A-L-F-R | 135 | M14 | this study |
| F2:A-:B- | 70ZU | 2010 | pig | C-S-A-L-F.G-R-CT-O | 70 | M24, oqxB | Liu,B.T. et al. |
| F2:A-:B- | Z39 | 2004 | pig | C-S-A-K-N-CT | 97 | M27, *qnrS,rmtB* | this study |
| F2:A-:B- | FS11Z2C | 2012 | pig | C-S-A-G-K-R-CT | 97 | M27*, rmtB* | this study |
| F2:A-:B- | FS044 | 2011 | pig | C-S-A-L-G-N-CT-F | 190 | M27, *rmtB* | this study |
| F2:A-:B- | S22 | 2004 | pig | C-S-A-T-G-K-N-R-CT | 97 | M27, *rmtB* | this study |
| F2:A-:B- | A62 | 2007 | avian | C-S-A-T-G-K-N-R-CT | 97 | M27, *rmtB* | Liu,B.T. et al. |
| F2:A-:B- | A64 | 2007 | avian | C-S-A-L-G-K-N-R-CT-O | 340 | M27*, rmtB, qnrB* | Liu,B.T. et al. |
| F2:A-:B- | A61 | 2007 | avian | C-S-A-G-K-N-R-CT-O | 97 | M27, *rmtB,qnrS,oqxB,qepA* | Liu,B.T. et al. |
| F2:A-:B- | S116 | 2007 | pig | C-S-A-L -F-T-R-O-CT | 80 | M27 | this study |
| F2:A-:B- | A83 | 2007 | avian | C-S-A-F-T-G-K-N-D-R-CT | 97 | M27*, rmtB* | this study |
| F2:A-:B- | A75 | 2007 | avian | C-S-A-R-CT-O | 97 | M27, *rmtB* | Liu,B.T. et al. |
| F2:A-:B- | A74 | 2007 | avian | C-S-A-L-G-K-N-R-CT | 194 | M27*, rmtB* | Liu,B.T. et al. |
| F2:A-:B- | A90 | 2005 | avian | C-S-R-CT-O | ND | M27, *oqxB,qepA,rmtB* | this study |
| F2:A-:B- | S87 | 2008 | pig | C-S-A-T-R-CT | 97 | M27 | this study |
| F2:A-:B- | 63 | 2010 | pet | C-S-A-L-F-K-R | 194 | M3, *rmtB,qepA* | Liu,B.T. et al. |
| F2:A-:B- | 43 | 2008 | pig | S-A-P | 97 | M55 | this study |
| F2:A-:B- | FS9Y2C | 2012 | avian | P-S-A-F-T-K-O | 194 | M55 | this study |
| F2:A-:B- | HHC12 | 2010 | avian | C-S-A-G-K-N-R-CT | 97 | M55 | this study |
| F2:A-:B- | TD41 | 2011 | pig | C-S-A-R | ND | M55 | this study |
| F2:A-:B- | FSD160 | 2009 | avian | C-S-A-G-K-N-R-CT | 194 | M55 | this study |
| F2:A-:B- | 5wei | 2010 | pig | N-C-S-A-L-F-O-K-G-N-CT | 100 | M65, *oqxB* | Liu,B.T. et al. |
| F2:A-:B- | A6 | 2007 | avian | C-S-A-T-R-CT | 70 | M65*, oqxB ,qnrS ,qepA* | Liu,B.T. et al. |
| F2:A-:B- | 7zu | 2010 | pig | C-S-A-G-K-R-CT-O | 80 | *oqxB,qepA* | Liu,B.T. et al. |
| F2:A-:B- | B19 | 2010 | pet | C-S-A-R-CT | 97 | *qnrB,qnrS,oqxB,qepA, rmtB* | this study |

Table S1. Continued.

| FAB^a^ | Isolate | Year | Source | Co-resistance^b^ | Size^c^ | Genes^d^ | Reference |
| --- | --- | --- | --- | --- | --- | --- | --- |
| F2:A-:B- | S76 | 2012 | pig | S-A-G-K | 190 | *rmtB,oqxB, qnrB* | this study |
| F2:A-:B- | A84 | 2007 | avian | C-S-A-G-N-R-CT | ND | *oqxB,qnrS* | this study |
| F2:A-:B- | A72 | 2007 | avian | C-S-A-F-G-K-N-R-CT-O | 97 | M27, *rmtB* | Liu,B.T. et al. |
| F2:A-:B10 | D4T10 | 2008 | pet | C-S-A-G-N-R-CT | 120 | M14 | this study |
| F2:A1:B- | TD157 | 2011 | pig | C-S-A-T-G-K-R-CT | 145 | M14 | this study |
| F2:A1:B- | B16 | 2010 | pet | N-C-S-A-T-F-G-K-N-D-R-CT | 120 | M14, *oqxB,qnrS* | this study |
| F2:A1:B- | BZC18 | 2009 | avian | C-S-A-R | ND | M55 | this study |
| F2:A1:B1 | S92 | 2012 | pig | N-C-S-A-L-G-K-N-R-CT | 145 | *rmtB* | this study |
| F2:A1:B1 | S93 | 2012 | pig | N-S-A-L-F-T-G-K-D-O | 194 | *rmtB*,*qnrS* | this study |
| F14:A-:B- | FS6J2C | 2012 | avian | C-S-A-N-R-G | ND | M14, *oqxB* | this study |
| F16:A-:B- | S53 | 2010 | pig | S-A-L-F-O | ND | *oqxB* | Liu,B.T. et al. |
| F16:A-:B- | A7 | 2007 | avian | S-N-O | 97 | *oqxB,qnrS* | this study |
| F16:A-:B- | Z13 | 2004 | avian | S-O | ND | *oqxB,qnrS* | this study |
| F16:A-:B1 | LZC4 | 2011 | pig | C-S-A-R | 60 | M55 | this study |
| F18:A-:B- | FS1J2G | 2012 | avian | C-S-A-K | 130 | M65 | this study |
| F18:A-:B- | FS12Y2G | 2012 | avian | C-S-A-T-G-N-R-CT | 145 | M55 | this study |
| F18:A-:B- | A44 | 2005 | avian | S-A-L-F-T-N-D-O | 145 | *oqxB* | this study |
| F18:A-:B8 | Z31 | 2004 | pig | N-S-A-T-G-N-O | 194 | *oqxB* | Liu,B.T. et al. |
| F18:A-:B1 | FS3Z3C | 2012 | pig | C-S-A-N-R-G-F | 145 | M55*, oqxB,rmtB* | Liu,B.T. et al. |
| F18:A-:B1 | FSD155 | 2009 | avian | C-S-A-L-R-G | ND | M55 | this study |
| F18:A-:B1 | 33-2 | 2010 | pet | C-S-A-N-R-G | 145 | M55*, oqxB,rmtB* | Liu,B.T. et al. |
| F18:A-:B1 | A26 | 2003 | avian | C-S-A-L-F-T-K-D-R | 145 | *oqxB* | Liu,B.T. et al. |
| F18:A-:B1 | A58 | 2004 | avian | S-A-L-T-G-N-D-O | 145 | *oqxB* | Liu,B.T. et al. |
| F18:A-:B1 | Z1 | 2004 | pig | S-A-L-T-N-D-O | 145 | *oqxB* | Liu,B.T. et al. |
| F18:A-:B1 | S106 | 2007 | avian | S-A-L-F-G-O | ND | *oqxB* | this study |
| F18:A-:B1 | FS12Y1C | 2012 | avian | N-S-A-L-T-K-D-O | 145 | M55 | this study |
| F18:A-:B1 | 32 | 2008 | pet | C-S-A-G | 194 | M14 | this study |
| F18:A-:B1 | LJC8 | 2010 | pig | C-S-A-R | ND | M55 | this study |
| F31:A4:B1 | FS336L | 2010 | pig | C-S-A-G-R | 190 | M15 | this study |
| F31:A4:B1 | FS340F | 2009 | pig | C-S-A-G | 194 | M15 | this study |
| F31:A4:B1 | FS345P | 2010 | pig | C-S-A-R | 194 | M15 | this study |
| F33:A-:B- | S104 | 2012 | pig | C-S-A-L-G-K-N-R-CT | 97 | M65 | this study |
| F33:A-:B- | B7 | 2010 | pet | C-S-A-G-K-N | 90 | *oqxB, rmtB* | this study |
| F33:A-:B- | S55-2 | 2012 | pig | C-S-A-T-G-K-N-R-CT-O | ND | *rmtB* | this study |
| F33:A-:B- | 85chang | 2010 | avian | C-S-A-F-G-K-N-R-CT-O | 170 | *oqxB, floR* | Liu,B.T. et al. |
| F33:A-:B- | FS6J1W | 2012 | avian | C-S-A-CT-G | ND | *oqxB* | this study |
| F33:A-:B- | S68 | 2010 | pig | S-A-L-F-G-O | ND | M65, *oqxB* | Liu,B.T. et al. |
| F33:A-:B- | FS11Z5 | 2012 | pig | C-S-A-T-G-K-N-R-CT | ND | M65, *rmtB* | this study |

Table S1. Continued.

| FAB^a^ | Isolate | Year | Source | Co-resistance^b^ | Size^c^ | Genes^d^ | Reference |
| --- | --- | --- | --- | --- | --- | --- | --- |
| F33:A-:B- | FS8J4C | 2012 | avian | C-S-A-R-CT | 97 | M65 | this study |
| F33:A-:B- | FS4Y2C | 2012 | avian | N-S-A-T-G-K-O | 145 | M14,*rmtB* | this study |
| F33:A-:B- | FS9Y1C | 2012 | avian | C-S-A-L-T-K-D-R- | 70 | M55,oqxB | Liu,B.T. et al. |
| F33:A-:B- | FS2Y1X | 2012 | avian | S-A-L-T-K-D-O- | 100 | M55,*oqxB* | Liu,B.T. et al. |
| F33:A-:B- | FS5E1D | 2012 | avian | C-S-A-L-F-K-G-N-CT-R | 100 | M55,*oqxB* | Liu,B.T. et al. |
| F33:A-:B- | 42-2 | 2010 | avian | C-S-A-L-F-T-N-R | 100 | M55,*oqxB* | Liu,B.T. et al. |
| F33:A-:B- | 2YC2 | 2011 | avian | C-S-A-L-F-K-G-N-CT-R | ND | M55,*rmtB* | this study |
| F33:A-:B- | FS13Z3C | 2012 | pig | N-C-S-A-F-K-T-G-N-R-CT | 120 | M55,*rmtB* | this study |
| F33:A-:B- | FS11Y2G | 2012 | avian | C-S-A-F-K-G-N-CT | 120 | M55,*rmtB* | this study |
| F33:A-:B- | FS6Y1G | 2012 | avian | C-A-L-F-R | 97 | M55,*rmtB* | this study |
| F33:A-:B- | 3-3-2 | 2008 | pig | C-S-A-N-R-CT | 60 | M55 | this study |
| F33:A-:B- | FS13Z2C | 2012 | pig | C-S-R-CT | ND | M55 | this study |
| F33:A-:B- | FS2Y3G | 2012 | avian | C-S-A-R-CT | 60 | M55 | this study |
| F33:A-:B- | FS5Z6D | 2012 | pig | C-S-A-K-G-R-CT | 97 | M55,*rmtB* | this study |
| F33:A-:B- | FS6Y4C | 2012 | avian | S-A-L-T-K-D-O- | 90 | M55,*rmtB* | this study |
| F33:A-:B- | FS11Z5F | 2012 | pig | C-S-A-K-G-CT-N | 90 | M55,*rmtB* | this study |
| F33:A-:B- | NND6 | 2010 | avian | C-S-A-R | 90 | M55 | this study |
| F33:A-:B- | FS1Z1X | 2012 | pig | C-S-A-G-K-R-CT | 90 | M55,*rmtB* | this study |
| F33:A-:B- | 38 | 2010 | pet | C-S-A-G-K-N-R-CT | 100 | M65,*rmtB* | Liu,B.T. et al. |
| F33:A-:B- | 50 | 2010 | pet | C-S-A-G-K-N-R-CT-O | 97 | M65,*rmtB* | Liu,B.T. et al. |
| F33:A-:B- | ping5-1 | 2008 | pig | C-S-A-G-K-N-R-CT | 97 | M65,*rmtB* | this study |
| F33:A-:B- | P161 | 2008 | pet | C-S-A-R-CT | 120 | M65,*rmtB* | this study |
| F33:A-:B- | 88 | 2010 | pig | C-S-A-G-K-R-CT-N | 97 | M65,*rmtB* | this study |
| F33:A-:B1 | 3YX1 | 2011 | avian | S-A-L-G-K-N-D-O | ND | *oqxB ,rmtB* | Liu,B.T. et al. |
| F33:A1:B- | LJC7 | 2010 | pig | C-S-A-G-K-R -CT | ND | M14 | this study |
| F35:A-:B- | C50 | 2008 | pet | C-S-G-K-R-CT | 97 | M14 | this study |
| F35:A-:B- | C41 | 2008 | pet | P-N-C-S-A-T-G-R | 97 | M14 | this study |
| F35:A-:B- | C34T11 | 2008 | pet | C-A-R-CT | ND | M14 | this study |
| F36:A1:B1 | A8 | 2010 | pet | C-S-A-G-N-R | 150 | M14 | this study |
| F43:A-:B- | NND5 | 2010 | avian | C-S-A-F-T-K-G-N-R-CT | 110 | M14, *qnrS* | this study |
| F43:A3:B | FSD158 | 2009 | avian | C-S-A-L-G-R | 145 | M55, *qnrS* | this study |
| F46:A-:B24 | 101Fei | 2010 | pig | S-A-L-F-G-O- | 130 | *oqxB* | Liu,B.T. et al. |
| F58:A-:B- | FS14J1 | 2012 | avian | C-S-A-R-CT | 145 | M65 | this study |

^a^ FAB, FII:FIA:FIB

^b^ A, ampicillin; S, streptomycin; M, amikacin; K, kanamycin; G, gentamycin; R, ceftriaxone; C, cefotaxime; CT, ceftiofur; N, nalidixic acid; P, ciprofloxacin; E, enrofloxacin; L, levofloxacin; O, olaquindox; T, tetracycline; H, chloramphenicol and F, florfenicol.

^c^ Size: kb

^d^ M55/14/27/65/15/3/24 is resistance genes abbreviations for M55_/14/27/65/15/3/24_, respectively.

ND, not determined.

**(a) (b)**


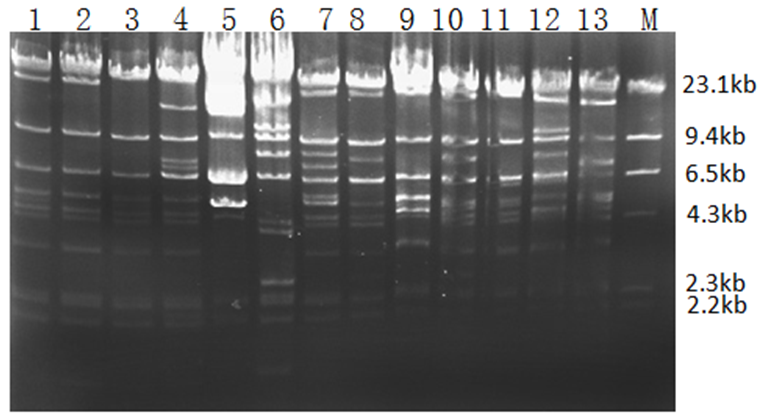

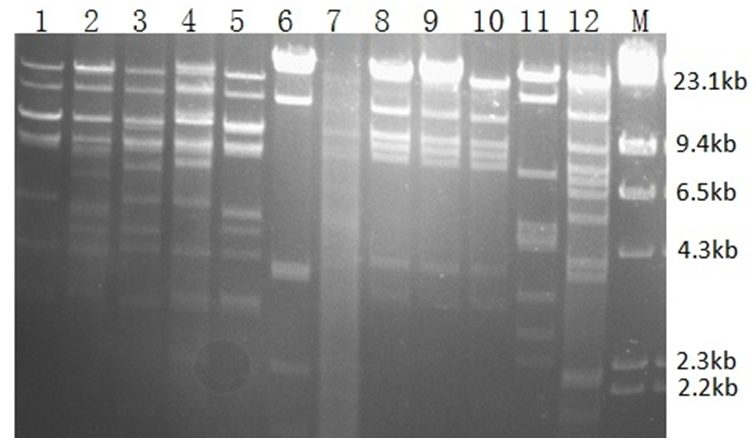


**Figure S1**. **RFLP analysis digested with *EcoRI* from IncF plasmids.**

Footnote: Lane M, λ-*Hind* III marker. IncF plasmids are labelled at the top: a) Lane1-FS2Y3G, Lane2-BZC18, Lane3-NND6, Lane4-FS4Y2C, Lane5-S53, Lane6-FS6J1W, Lane7-FS13Z2C, Lane8-FS1Z1X, Lane9-38, Lane10-FS6Y1G, Lane11-FS11Z5F, Lane12-S55, Lane13-FS8J4C.

b) Lane1-FS12Y2G, Lane2-FS3Z3C, Lane3-FS1J2G, Lane4-33-2, Lane5-FSD155, Lane6-LJC8, Lane7-32, Lane8-FS336L, Lane9- FS336F, Lane10- FS336P, Lane11-FS5E6D, Lane12- FS14J1.

Figure S2. Electrophoresis result of p42-2 digested with *EcoR*I obtained using DNAstar. LamBBX: lambda BstEII, BgIII, XhoI marker; LamH3: lambda HindIII marker; *EcoRI*: p42-2digested with *EcoR*I
